# CME‐1, a novel polysaccharide, suppresses iNOS expression in lipopolysaccharide‐stimulated macrophages through ceramide‐initiated protein phosphatase 2A activation

**DOI:** 10.1111/jcmm.13424

**Published:** 2017-12-07

**Authors:** Joen‐Rong Sheu, Zhih‐Cherng Chen, Ming‐Jen Hsu, Shwu‐Huey Wang, Kuo‐Wei Jung, Wei‐Fan Wu, Szu‐Han Pan, Ruei‐Dun Teng, Chih‐Hao Yang, Cheng‐Ying Hsieh

**Affiliations:** ^1^ Department of Pharmacology School of Medicine Taipei Medical University Taipei Taiwan; ^2^ Graduate Institute of Medical Sciences School of Medicine Taipei Medical University Taipei Taiwan; ^3^ Department of Cardiology Chi‐Mei Medical Center Tainan City Taiwan; ^4^ Department of Pharmacy Chia Nan University of Pharmacy & Science Tainan City Taiwan; ^5^ Core Facility Center Office of Research and Development Taipei Medical University Taipei Taiwan

**Keywords:** CME‐1, immunomodulatory property, protein phosphatase 2A, ceramide, reactive oxygen species

## Abstract

CME‐1, a novel water‐soluble polysaccharide purified from *Ophiocordyceps sinensis* mycelia, has anti‐oxidative, antithrombotic and antitumour properties. In this study, other major attributes of CME‐1, namely anti‐inflammatory and immunomodulatory properties, were investigated. Treating lipopolysaccharide (LPS)‐stimulated RAW 264.7 cells with CME‐1 concentration‐dependently suppressed nitric oxide formation and inducible nitric oxide synthase (iNOS) expression. In the CME‐1‐treated RAW 264.7 cells, LPS‐induced IκBα degradation and the phosphorylation of p65, Akt and mitogen‐activated protein kinases (MAPKs), including extracellular signal‐regulated kinase, c‐Jun N‐terminal kinase and p38, were reduced. Treatment with a protein phosphatase 2A (PP2A)‐specific inhibitor, significantly reversed the CME‐1‐suppressed iNOS expression; IκBα degradation; and p65, Akt and MAPK phosphorylation. PP2A activity up‐regulation and PP2A demethylation reduction were also observed in the cells. Moreover, CME‐1‐induced PP2A activation and its subsequent suppression of LPS‐activated RAW 264.7 cells were diminished by the inhibition of ceramide signals. LPS‐induced reactive oxygen species (ROS) and hydroxyl radical formation were eliminated by treating RAW 264.7 cells with CME‐1. Furthermore, the role of ceramide signalling pathway and anti‐oxidative property were also demonstrated in CME‐1‐mediated inhibition of LPS‐activated primary peritoneal macrophages. In conclusion, CME‐1 suppressed iNOS expression by up‐regulating ceramide‐induced PP2A activation and reducing ROS production in LPS‐stimulated macrophages. CME‐1 is a potential therapeutic agent for treating inflammatory diseases.

## Introduction

Inflammation is a defence mechanism triggered by the innate immune system that responds to invasive microbes and tissue injury. In response to pathogen‐ and environment‐derived stimuli, innate immune cells are stimulated to produce pro‐inflammatory cytokines, such as tumour necrosis factor‐α and interleukins, and generate nitric oxide (NO) from inducible nitric oxide synthase (iNOS) 1, 2. Macrophages are crucial immune cells that are stimulated by receptors that recognize microbial products and elicit inflammatory signalling cascades 3. Lipopolysaccharide (LPS) is a representative pathogen‐associated molecular pattern and a cell wall component of gram‐negative bacteria. LPS is considered the most potent immunostimulant among all bacterial cell wall components 4. Stimulating macrophages with LPS induces various inflammatory signalling cascades by activating toll‐like receptor 4 (TLR4) complexes. The interaction recruits proteins including myeloid differentiation primary response gene 88 and interleukin‐1 receptor‐associated kinases and stimulates tumour necrosis factor receptor‐associated factor 6, which subsequently activates nuclear factor (NF)‐κB and AP‐1 5. Additionally, mitogen‐activated protein kinases (MAPKs) are central signalling cascades involved in TLR4‐activated signalling events 6. Activating these inflammation‐related transcription factors induces the synthesis of pro‐inflammatory mediators and the expression of surface molecular proteins, which are involved in cell recruitment and activation 5. Despite the importance of inflammation in innate immunity, excess inflammatory responses cause pathological conditions such as rheumatoid arthritis and sepsis. Consequently, developing new therapeutic agents for anti‐inflammatory therapy is essential for the therapeutic control of inflammatory diseases 7.

**Figure 1:**
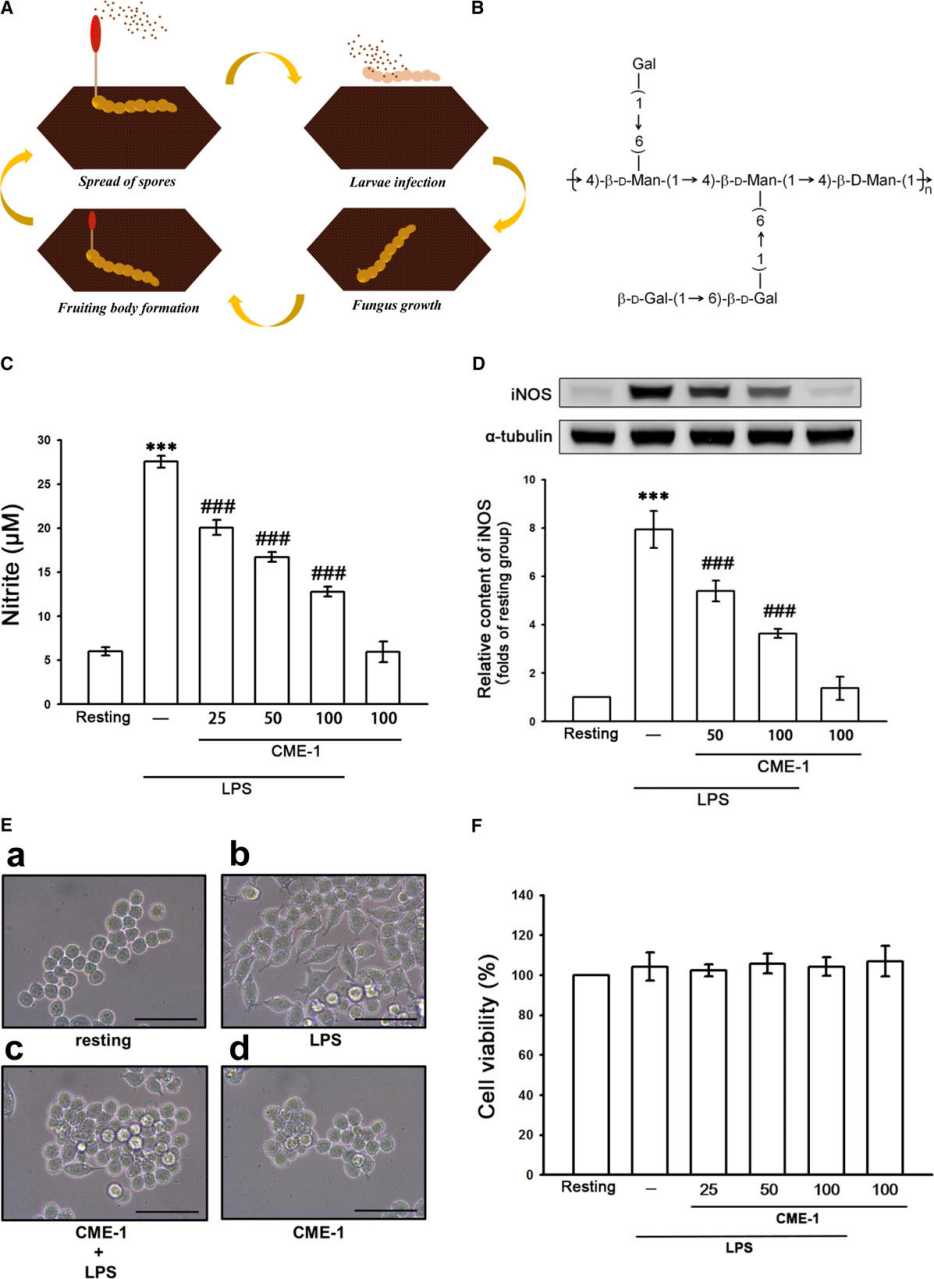
Life cycle of *Ophiocordyceps sinensis* (**A**), chemical structure of CME‐1 (**B**) and effects of CME‐1 on nitric oxide production, iNOS expression, morphological changes and cell viability in lipopolysaccharide (LPS)‐stimulated RAW 264.7 cells. RAW 264.7 cells were treated with PBS (resting group) or pre‐treated with CME‐1 (25–100 μg/ml) for 20 min and then treated with LPS (1 μg/ml) for 24 hrs. (**C**) Nitrite concentration, (**D**) iNOS protein level and (**F**) cell viability were evaluated as described in the Methods. Data are presented as the mean ± S.E.M. (*n *=* *3). (**E**) Cell morphology was observed using optical microscopy. Black bar = 50 μm; ***P < 0.001, compared with the resting group; ^###^ *P < *0.001, compared with the LPS group.

*Ophiocordyceps* is a genus of fungi that grows on the larvae of insects that have been infected by *Ophiocordyceps sinensis* (*O. sinensis*) (Fig. 1A). *Ophiocordyceps sinensis* has been widely used to treat immunological diseases, tumour growth, kidney diseases and inflammatory conditions 8, 9. *Ophiocordyceps sinensis* mycelium (OM) extract is a promising source of therapeutic agents because it can be purified for mass production. Additionally, the amount of polyphenols and bioactive components is higher in OM than in the fruiting body of *O*. *sinensis* 9. Numerous bioactive compounds, including sterols, cordycepin, polyphenols and polysaccharides, have been found in *Ophiocordyceps* species. Among these bioactive compounds, polysaccharides are the major antioxidants and have anti‐inflammatory, anticancer and immunomodulatory effects 10. CME‐1, a novel, water‐soluble, 27.6‐kD polysaccharide, was purified from OM, and it contained mannose and galactose in a ratio of 4:6 (Fig. 1B). Wang *et al*. 11 discovered that CME‐1 can protect macrophages by reducing oxidative stress. We recently demonstrated that CME‐1 has potent antithrombotic and antitumour properties 12, 13.

However, the anti‐inflammatory effect of CME‐1, a major therapeutic activity of *O. sinensis*, has not been thoroughly investigated. This study examined the detailed signalling pathway associated with the CME‐1‐mediated inhibition of iNOS expression in LPS‐stimulated macrophages.

## Materials and Methods

### Materials

Foetal calf serum (FCS), Dulbecco's modified Eagle medium (DMEM), l‐glutamine penicillin/streptomycin and trypsin (0.25%) were purchased from Invitrogen (Thermo Fisher Scientific, Waltham, MA, USA). LPS (*Escherichia coli* 0127:B8), 3‐(4,5‐dimethylthiazol‐2‐yl)‐2,5‐diphenyltetrazolium bromide (MTT), Brewer thioglycollate medium and 2′,7′‐dichlorofluorescein diacetate (DCFDA) were purchased from Sigma‐Aldrich (St. Louis, MO, USA). Okadaic acid (OA) (>98% purity) was purchased from Merck Millipore (Billerica, MA, USA). 3‐OMe‐SM was purchased from Biomol (Plymouth Meeting, PA, USA).

The anti‐dimethyl‐protein phosphatase 2A (deM‐PP2A) and anti‐p65 monoclonal antibodies (mAbs), and anti‐iNOS polyclonal antibody (pAb) were purchased from Santa Cruz Biotechnology (Dallas, TX, USA). The anti‐phospho‐p65 (Ser536), anti‐phospho‐Akt (Ser473), anti‐JNK, anti‐phospho‐c‐JNK (Thr183/Tyr185), anti‐phospho‐p44/p42 ERK (Thr202/Tyr204), and anti‐phospho‐p38 MAPK (Thr180/Tyr182) pAbs, and anti‐IκBα, anti‐Akt, anti‐ERK and anti‐p38 MAPK mAbs were purchased from Cell Signaling (Danvers, MA, USA). The anti‐PP2A pAb was purchased from GeneTex (Irvine, CA, USA). The anti‐α‐tubulin mAb was from Thermo Fisher Scientific. The horseradish peroxidase (HRP)‐conjugated donkey anti‐rabbit IgG pAb, and sheep antimouse IgG pAb, the Western blotting detection reagent of enhanced chemiluminescence (ECL) and Hybond^®^‐P polyvinylidene difluoride (PVDF) blotting membranes were purchased from GE Healthcare Life Sciences (Waukesha, WI, USA). A PP2A immunoprecipitation phosphatase assay kit was purchased from Merck Millipore.

### Cell cultivation

RAW264.7 cell, a cell line of murine macrophage, was purchased from ATCC (ATCC number: TIB‐71). The cells were maintained in DMEM supplemented with 10% FCS and penicillin G (100 units/ml)/streptomycin (100 mg/ml) at 37°C in a humidified atmosphere of 5% CO_2_/95% air.

### Extraction of CME‐1 from *Ophiocordyceps sinensis* mycelia

CME‐1 was purified as Wang *et al*. described 11. Dried OM powder (200 g) was extracted by double‐distilled water (DD water) (200 ml) for three times (3 hrs, 25 °C). These extracts were collected and then concentrated to obtain a water‐soluble fraction of OM (65 g, 33% of crude residues). The OM extraction (2 g) was applied to Sephacryl G‐15 column (2.5 × 45 cm) and eluted by DD water to yield CME‐1 (12% of OM extraction). CME‐1 was determined and collected at the absorbance of 490 nm by sulphuric acid–phenol method. In addition, the CME‐1 fraction was hydrolysed in trifluoroacetic acid (8 hrs, 112 ℃). The hydrolysate was eluted through a CarboPac PA10 column (2 × 250 mm) by a mixture of 200 mM NaOH and DD water (the ratio of volume is 8:92). The composition of CME‐1 was identified using high‐performance anion‐exchange chromatography with pulsed amperometric detection (ICS‐3000 ion chromatography system; Dionex, Sunnyvale, CA, USA). We next analysed the molecular weight of CME‐1 by diffusion‐ordered NMR spectroscopy (AV600 NMR spectrometer; Bruker, Rheinstetten, Germany) 14, and the chemical structure was determined by a GCMS profile as described previously 15.

### Cell viability assay

2 × 10^5^ RAW 264.7 cells per well were seeded with DMEM containing 10% FCS on 24‐well culture plates for 24 hrs. The cells were pre‐treated with CME‐1 (25, 50 and 100 μg/ml) or an isovolumetric solvent control (PBS) for 20 min. and then stimulated with LPS (1 μg/ml) or left unstimulated for 24 hrs. Cell viability was measured using MTT assay to evaluate the ability of mitochondria in cells 16. The cell viability index was calculated as follows: (absorbance of treated cells ÷ absorbance of control cells) × 100%.

### Determination of nitric oxide production

To determine nitric oxide production, the level of nitrite/nitrate, stable oxidative end products of nitric oxide, was measured as previously described 17 with minor modifications. 8 × 10^5^ RAW 264.7 cells were seeded on 6‐cm dishes with DMEM containing 10% FCS for 24 hrs. The cells were pre‐treated with CME‐1 (25, 50 and 100 μg/ml) or PBS for 20 min. and then stimulated with LPS (1 μg/ml) or left unstimulated for 24 hrs. These conditioned media were collected for the analysis. The level of nitrite/nitrate was determined by Griess test (1% sulphanilamide and 0.1% naphthalenediamine dissolved in 2.5% phosphoric acid). The absorbance of samples was determined at 550 nm by a MRX absorbance reader (Dynex Technologies, Chantilly, VA, USA). The concentrations of nitrite/nitrate were calculated by a standard curve performed through the linear regression of absorbance measurements of standard solutions (sodium nitrite dissolved in the same culture medium).

### Western blotting assay

Western blotting assay was performed to determine the protein expression in cells as previously described 17. RAW 264.7 cells (8 × 10^5^ cells/dish) were seeded on 6‐cm dishes with DMEM containing 10% FCS for 24 hrs. The cells were pre‐treated with CME‐1 (25, 50 and 100 μg/ml) or PBS for 20 min. and then stimulated with LPS (1 μg/ml) or left unstimulated according to the experimental design. Subsequently, the cellular proteins were extracted with a lysis buffer and prepared with sodium dodecyl sulphate (SDS) loading dye. The prepared protein samples (50 μg) were applied to SDS‐polyacrylamide gel for the electrophoresis, and the separated protein bands were then transferred on PVDF membranes (0.45 μm) electrophoretically. The membranes were blocked with 5% skim milk dissolved in TBST buffer (10 mM Tris‐base, 100 mM NaCl and 0.01% Tween 20) for 1 hr and then recognized with different primary antibodies before incubated with secondary antibody for 1 hr. The ECL system was used to detect the immune‐reactive bands in this study. The densitometry of protein bands was performed by Biolight Windows Application, V2000.01 (Bio‐Profil, Vilber Lourmat, France).

### Determination of PP2A activity

An immunoprecipitation phosphatase assay kit which manufactured by EMD Millipore (Temecula, CA, USA) was used to measure PP2A activity. Based on the manufacturer's instructions, cellular proteins (200 μg) were immunoprecipitated with an anti‐PP2A catalytic subunit antibody, and the immunoprecipitated PP2A was then reacted with the substrate (750 μM phosphoprotein, amino acid sequence KRpTIRR) in a protein phosphatase assay buffer for 10 min. at room temperature. Reactions were terminated by adding a malachite green solution (100 μl). The absorbance was determined by a microplate reader at 650 nm.

### Quantification of ceramide species

The quantification of ceramides in macrophages by liquid chromatography coupled with tandem mass spectrometry (LC/MS/MS) was performed as described previously 11, 17 with some modifications. Briefly, raw 264.7 cells were treated according to the experimental design and then washed twice by ice‐cold PBS and collected in 0.5 ml of PBS. The procedure of extraction and quantification of ceramides was performed as previously described 11, 17. In this study, the precursor to product ion transitions were m/z 538.5.8 → 264.5 for C16:0 ceramide, m/z 566.5 → 264.5 for C18:0 ceramide, m/z 622.6 → 264.5 for C22:0 ceramide, m/z 650.6 → 264.5 for C24:0 ceramide, m/z 648.6 → 264.5 for C24:1 ceramide and m/z 552.5 → 264.5 for C17:0 ceramide for the multiple reaction‐monitoring with a well time of 15 ms. Analyst software version 1.4.2 (Applied Biosystems, Carlsbad, CA, USA) was used to measure the concentrations of unknowns, calibration standards and quality controls. Total ceramide was the sum of ceramide subspecies including C16:0, C18:0, C22:0, C24:0 and C24:1.

### Evaluation of intracellular reactive oxygen species (ROS) by flow cytometry

RAW 264.7 cells (5 × 10^5^ cells/Eppendorf tube) were loaded with 20 μM DCFDA for 20 min. After treatment with CME‐1 (100 μg/ml) or PBS for 20 min., cells were then stimulated by the addition of LPS (1 μg/ml) or left unstimulated according to experimental design. The cells were washed with PBS and detached using trypsin. The content of intracellular ROS was evaluated by flow cytometric assay (Beckman Coulter, Brea, CA, USA). The data of each group were collected from 1 × 10^4^ cells.

### Electron spin resonance (ESR) spectrometry for the measurement of hydroxyl radical formation

The ESR method involved using a Bruker EMX ESR spectrometer (Billerica, MA, USA) as described previously 16 with some modifications. RAW 264.7 cells (5 × 10^5^ cells/Eppendorf tube) were pre‐treated with CME‐1 (100 μg/ml) or PBS for 20 min., and LPS (1 μg/ml) was subsequently added. ESR spectra were recorded by using a quartz flat cell designed for aqueous solutions. The experimental condition was performed by 20 mW of power at 9.78 GHz, with a scan range of 100 G, and the receiver gain was 5 × 10^4^.

### Isolation of primary peritoneal macrophages

Male C57BL/6 mice (age, 6 weeks) were utilized to isolate primary peritoneal macrophages as described previously 18 with some modifications. All animal experiments and care procedures conformed to the Guide for the Care and Use of Laboratory Animals (NIH publication no. 85–23, 1996) and were approved by the Institutional Animal Care and Use Committee of Taipei Medical University. Each mouse was injected 2 ml of 4% (w/v) Brewer thioglycollate medium into the peritoneal cavity for three days. The mouse was then killed and injected 5 ml of ice‐cold PBS (contained 3% FCS) into the peritoneal cavity. After injection and gently massage of the peritoneum, the peritoneal macrophages were collected in tubes. The above procedure was repeated for three times. These collected cell suspensions were centrifuged at 400 *g*; after discarding the supernatant, the cells were re‐suspended in DMEM for the experiments.

### Statistical analysis

The results are presented as the mean ± standard error (S.E.M.) and the number of observations (*n*). First, data were assessed using one‐way analysis of variance (one‐way anova). If one‐way anova revealed significant differences among the group means, a subsequent comparison of Newman–Keuls method was performed. A *P* value <0.05 was indicated a statistically significant difference.

## Results

### Effects of CME‐1 on nitric oxide production and iNOS expression in RAW 264.7 cells stimulated by LPS

Figure 1C illustrates that LPS treatment (1 μg/ml) of RAW 264.7 cells for 24 hrs increased nitric oxide production from 6.0 ± 0.2 to 27.6 ± 0.3 μM (*P *<* *0.001; *n *=* *3). CME‐1 treatment (20, 50 and 100 μg/ml) concentration‐dependently inhibited nitric oxide production by 34.3%, 50.0% and 68.5%, respectively, in LPS‐stimulated RAW 264.7 cells. We then determined whether the expression of iNOS protein, which catalyses nitric oxide generation, is also inhibited by CME‐1 in RAW 264.7 cells activated by LPS. Compared with the control group, LPS treatment (1 μg/ml) significantly increased iNOS expression in the treated group, whereas CME‐1 treatment (50 and 100 μg/ml) concentration‐dependently inhibited iNOS expression in LPS‐stimulated RAW 264.7 cells (*P *<* *0.001, *n *=* *3; Fig. 1D). These results suggest that the CME‐1‐mediated inhibition of nitric oxide formation results from the down‐regulation of iNOS expression in RAW 264.7 cells stimulated by LPS. In addition, the cell morphology of RAW 264.7 cells treated with CME‐1 (100 μg/ml) in the presence or absence of LPS (1 μg/ml) was observed using microscopy. Unstimulated RAW 264.7 cells exhibited a round morphology, whereas LPS‐stimulated RAW 264.7 cells exhibited an irregular morphology, with pseudopodia formation and cell spreading. CME‐1 treatment reversed this morphology change in LPS‐stimulated RAW 264.7 cells (Fig. 1E).

To examine whether the pharmacological activity or cytotoxicity of CME‐1 inhibits nitric oxide production, the viability of CME‐1‐treated RAW 264.7 cells was determined using a MTT assay. CME‐1 treatment (20, 50 and 100 μg/ml) did not exert significant cytotoxic effects on LPS‐stimulated RAW 264.7 cells (Fig. 1F).

### Inhibition of CME‐1 on the NF‐κB, Akt and MAPK signalling pathways in RAW 264.7 cells stimulated by LPS

**Figure 2:**
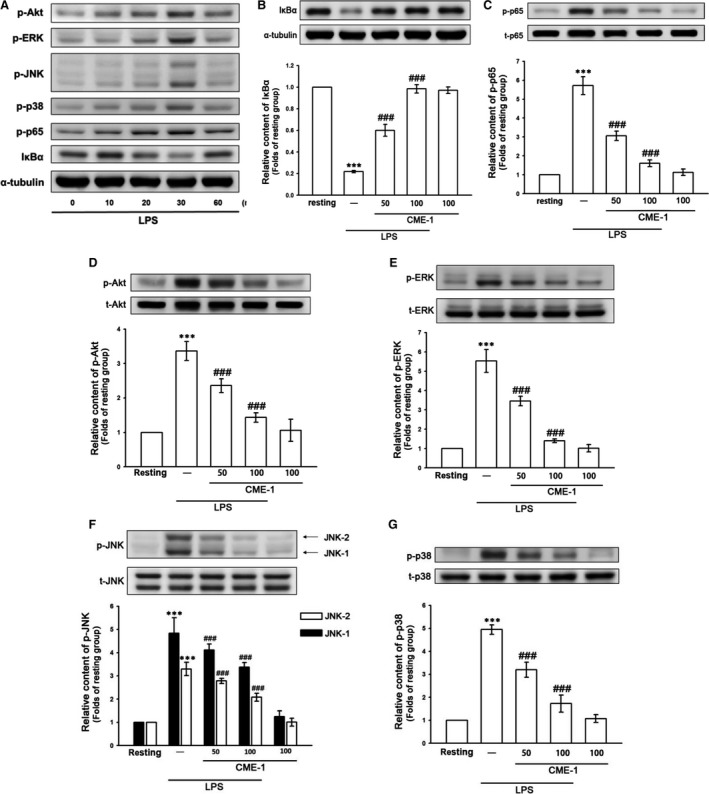
Time‐course analysis of lipopolysaccharide (LPS)‐activated signalling molecules and effects of CME‐1 on LPS‐induced IκBα degradation and the phosphorylation of p65, Akt, ERK, JNK and p38 in RAW 264.7 cells. (**A**) RAW 264.7 cells were treated with LPS (1 μg/ml) for the indicated time. IκBα degradation and p65, Akt, ERK, JNK and p38 phosphorylation were determined using an immunoblotting assay as described in the Methods. (**B**–**G**) RAW 264.7 cells were treated with PBS (resting group) or CME‐1 (25–100 μg/ml) for 20 min, followed by LPS (1 μg/ml) for 30 min. IκBα degradation and the phosphorylation of p65, Akt, ERK, JNK and p38 were evaluated using immunoblotting. Data are presented as the mean ± S.E.M. (*n *=* *3). ***P < 0.001, compared with the resting group; ^###^ *P < *0.001, compared with the LPS group.

To elucidate the mechanism of CME‐1‐mediated inhibition of iNOS expression in macrophages activated by LPS, we examined the effects of CME‐1 on the major pro‐inflammatory signal transduction pathways of the innate immune system, including the NF‐κB, Akt and MAPK signalling cascades 5, 6, 19. We first performed a time‐course analysis of activation of these signalling molecules in LPS‐stimulated RAW 264.7 cells. Akt, MAPK and p65 phosphorylation and IκBα degradation peaked at 30 min. after LPS treatment (1 μg/ml) (Fig. 2A). Therefore, we used this time‐point for subsequent experiments.

CME‐1 treatment (50 and 100 μg/ml) concentration‐dependently reversed IκBα degradation and inhibited p65, Akt, extracellular signal‐regulated kinase (ERK), c‐Jun N‐terminal kinase (JNK) and p38 phosphorylation in LPS‐stimulated RAW 264.7 cells (Fig. 2B–G). Thus, CME‐1 treatment comprehensively suppressed these pro‐inflammatory signalling pathways in LPS‐stimulated RAW 264.7 cells.

### Role of protein phosphatase 2A in the inhibitory effects of CME‐1 in RAW 264.7 cells stimulated by LPS

**Figure 3:**
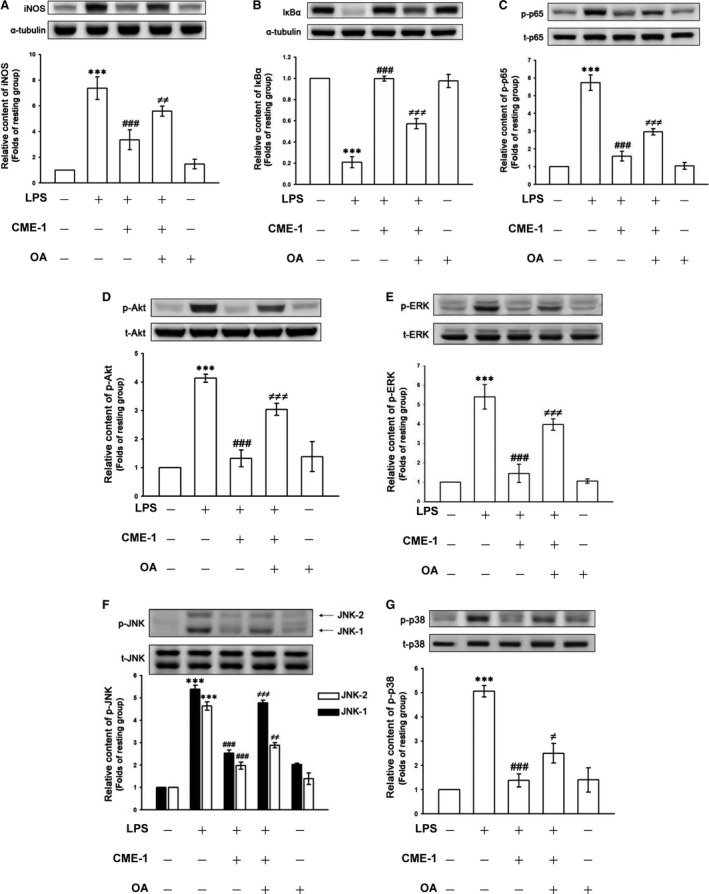
Role of protein phosphatase 2A (PP2A) in CME‐1‐inhibited iNOS expression, IκBα degradation and the phosphorylation of p65, Akt, ERK, JNK and p38 in lipopolysaccharide (LPS)‐stimulated RAW 264.7 cells. RAW 264.7 cells were pre‐treated with 10 nM OA and then treated with CME‐1 (100 μg/ml). LPS (1 μg/ml) was then added. (**A**) iNOS expression; (**B**) IκBα degradation; and (**C**) p65, (**D**) Akt, (**E**) ERK, (**F**) JNK and (**G**) p38 phosphorylation were determined using immunoblotting as described in the Methods. Data are presented as the mean ± S.E.M. (*n *=* *3). ***P < 0.001, compared with the resting group; ^###^ *P < *0.001, compared with the LPS group; ^≠^ *P *<* *0.05, ^≠≠^ *P < *0.01 and ^≠≠≠^ *P < *0.001, compared with the LPS + CME‐1 group.

Protein kinase phosphorylation was known to be regulated by protein phosphatases 20. We examined whether PP2A, a major mammalian protein phosphatase 21, is involved in CME‐1‐mediated comprehensive inhibition of LPS‐stimulated signalling pathways in RAW 264.7 cells. We used OA, a specific PP2A inhibitor 22, to determine the role of PP2A in the inhibitory mechanisms of CME‐1. The inhibitory effects of CME‐1 on iNOS expression; IκBα degradation; and p65, Akt, ERK, JNK and p38 phosphorylation were all significantly reversed after OA pre‐treatment (10 nM) of LPS‐stimulated RAW 264.7 cells (Fig. 3A–G). The results indicate a critical role of PP2A in the inhibitory mechanisms of CME‐1 in LPS‐stimulated RAW 264.7 cells.

**Figure 4:**
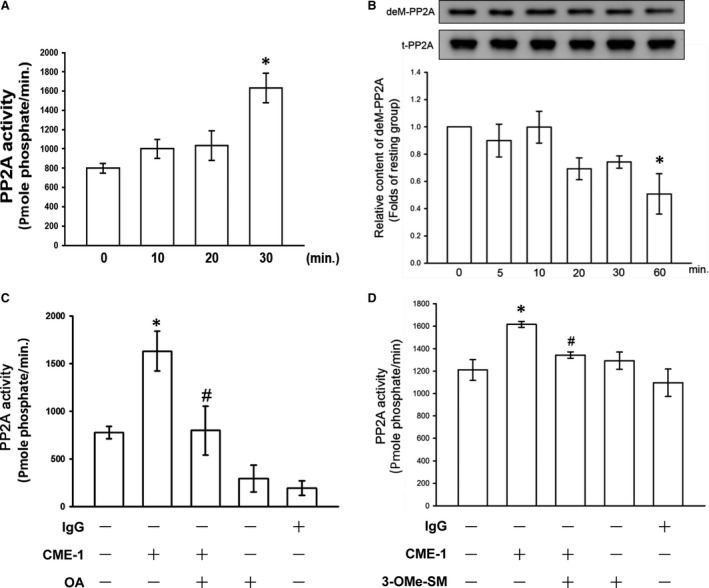
Effects of CME‐1 on protein phosphatase 2A (PP2A) activation in RAW 264.7 cells. RAW 264.7 cells were treated with CME‐1 (100 μg/ml) for the indicated time. (**A**) PP2A activity and (**B**) the dimethyl‐PP2A (deM‐PP2A) protein level were evaluated as described in the Methods. RAW 264.7 cells were pre‐treated with (**C**) 10 nM OA or (**D**) 30 μM 3‐OMe‐SM and then treated with CME‐1 (100 μg/ml) for 30 min. Data are presented as the mean ± S.E.M. (*n *=* *3). *P < 0.05, compared with the untreated group; ^#^ *P < *0.05, compared with the CME‐1 group

Next, we directly determined the effects of CME‐1 on PP2A activity in RAW 264.7 cells. CME‐1 treatment (100 μg/ml) significantly increased PP2A activity and reduced dimethyl‐PP2A expression in RAW 264.7 cells (Fig. 4A and B). Moreover, CME‐1‐induced PP2A activation was significantly abolished by OA pre‐treatment of the RAW 264.7 cells (Fig. 4C).

### Ceramide signalling in the inhibitory mechanisms of CME‐1 in LPS‐stimulated RAW 264.7 cells

**Figure 5:**
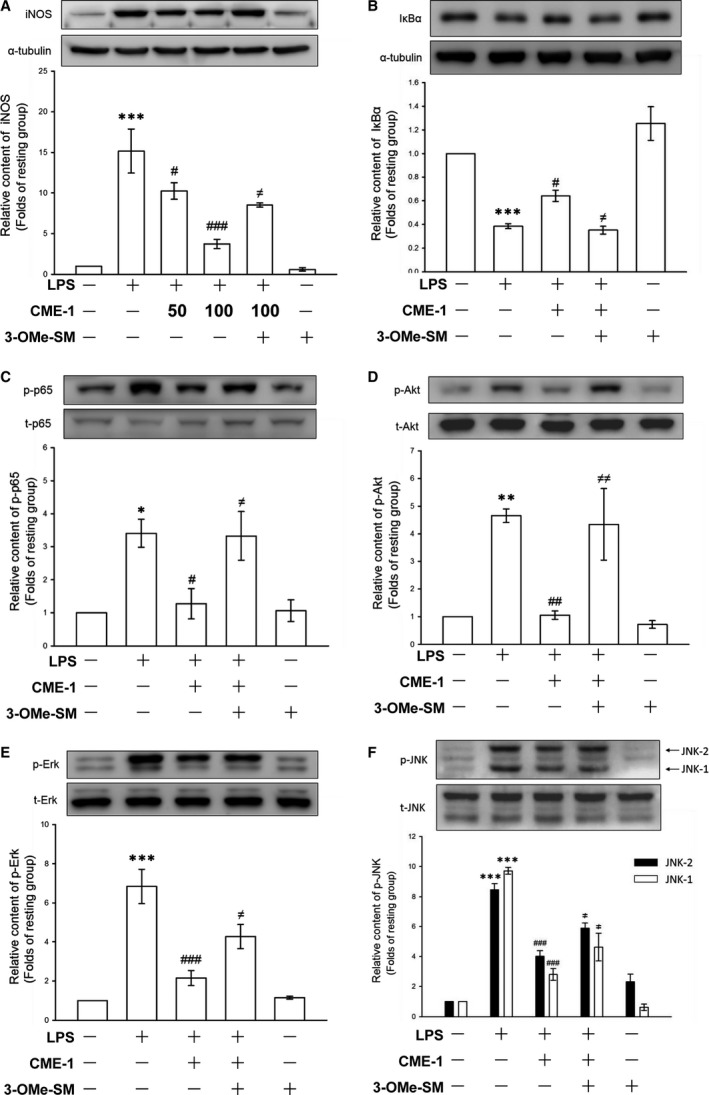
Effects of 3‐OMe‐SM on CME‐1‐inhibited iNOS expression, IκBα degradation and the phosphorylation of p65, Akt, ERK and JNK in lipopolysaccharide (LPS)‐stimulated RAW 264.7 cells. RAW 264.7 cells were pre‐treated with 30 μM 3‐OMe‐SM, a specific neutral sphingomyelinase inhibitor, and then treated with CME‐1 (100 μg/ml), followed by LPS (1 μg/ml). (**A**) iNOS expression; (**B**) IκBα degradation; and (**C**) p65, (**D**) Akt, (**E**) ERK and (**F**) JNK phosphorylation were determined using immunoblotting as described in the Methods. Data are presented as the mean ± S.E.M. (*n *=* *3). *P <* *0.05, **P <* *0.01 and ***P < 0.001, compared with the resting group; ^#^ *P *<* *0.05, ^##^ *P *<* *0.01 and ^###^ *P < *0.001, compared with the LPS group; ^≠^ *P *<* *0.05 and ^≠≠^ *P < *0.01 compared with the LPS + CME‐1 group.

**Figure 6:**
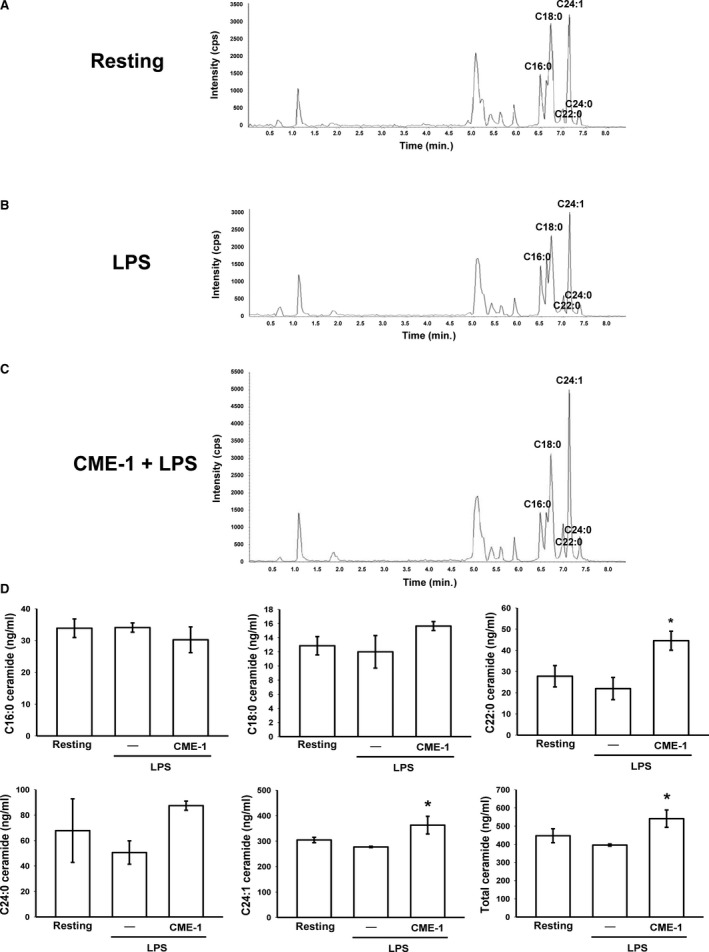
CME‐1‐increased ceramide formation in lipopolysaccharide (LPS)‐activated RAW264.7 cells. RAW 264.7 cells were treated with (**A**) PBS (resting group), (**B**) LPS (1 μg/ml) or (**C**) LPS + CME‐1 (100 μg/ml) for 20 min. (**D**) Intracellular C16‐, C18‐, C22‐, C24‐ and C24:1‐ceramides were quantified in RAW264.7 cells using LC/MS/MS as described in the Methods. Data are presented as the mean ± S.E.M. (*n *=* *3). *P <* *0.05, compared with the LPS group.

Ceramide was first described as an endogenous activator for PP2A in 1993 23. We suppressed ceramide formation using 3‐O‐methyl‐sphingomyelin (3‐OMe‐SM), a specific neutral sphingomyelinase inhibitor, and determined the role of ceramide signalling in the inhibitory mechanisms of CME‐1 in LPS‐stimulated RAW 264.7 cells. As illustrated in Figure 4D, pre‐treating RAW 264.7 cells with 3‐OMe‐SM (30 μM) significantly inhibited CME‐1‐increased PP2A activation. In addition, CME‐1‐suppressed iNOS expression; IκBα degradation; and p65, Akt and MAPK phosphorylation were reversed in LPS‐stimulated RAW 264.7 cells (Fig. 5). Furthermore, as shown in Figure 6, treatment of CME‐1 (100 μg/ml) significantly increased ceramide formation in RAW 264.7 cells activated by LPS. The cytosolic content of C22:0, C24:1 and sum of ceramides was increased in the LPS + CME‐1 group compared to the LPS group (*P *<* *0.05, *n *=* *3).

### Effects of CME‐1 on ROS formation in LPS‐activated RAW 264.7 cells

**Figure 7:**
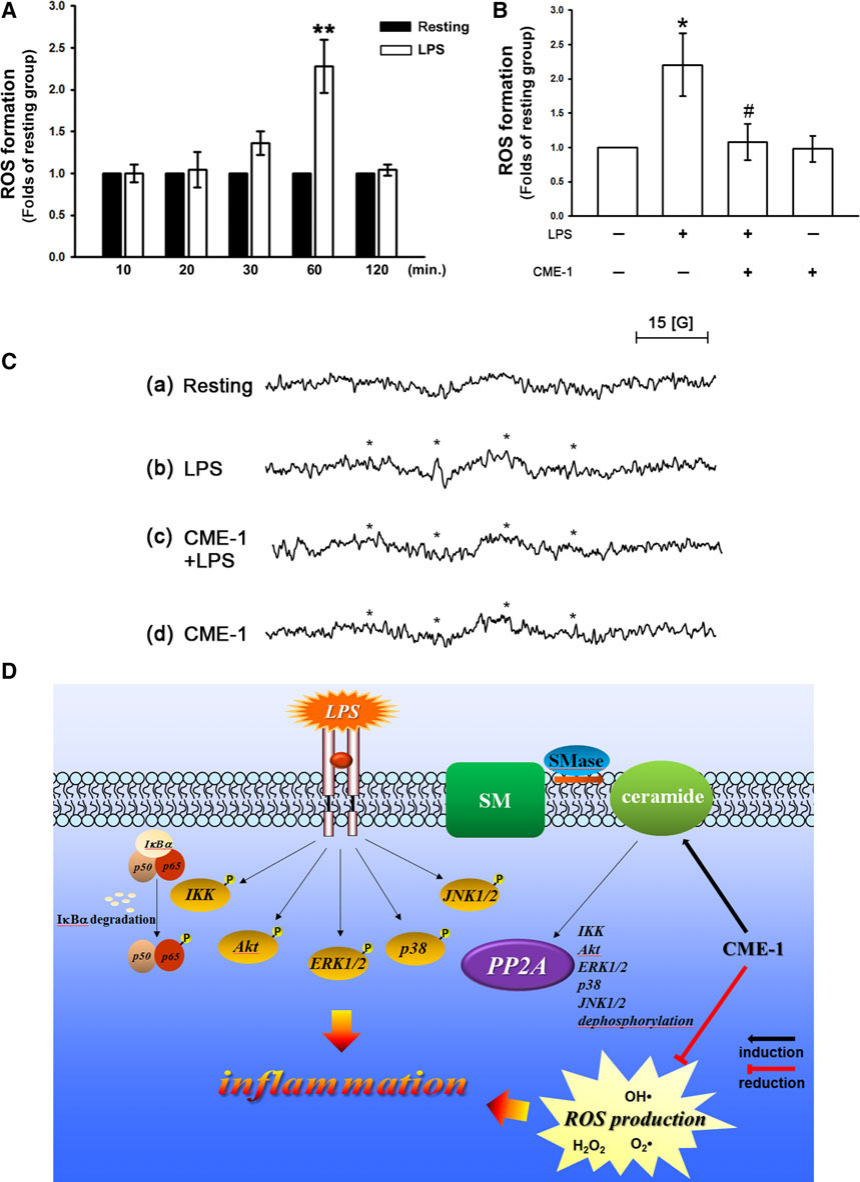
Effects of CME‐1 on reactive oxygen species (ROS) and hydroxyl radical production and hypothetical inhibitory mechanism of CME‐1‐mediated effects (**D**) in lipopolysaccharide (LPS)‐stimulated RAW 264.7 cells. (**A** and **B**) RAW 264.7 cells (5 × 10^5^ cells/Eppendorf tube) were treated with or without CME‐1 (100 μg/ml) for 20 min. followed by LPS (1 μg/ml) for the indicated periods to trigger ROS formation. Intracellular ROS content was determined using a DCFDA assay as described in the Methods. Data are presented as the mean ± S.E.M. (*n *=* *3). *P <* *0.05 and **P <* *0.01, compared with the control group; ^#^ *P *<* *0.05, compared with the LPS group. (**C**) RAW 264.7 cells (5 × 10^5^ cells/Eppendorf tube) were treated with PBS (trace a and b) or CME‐1 (trace c and d, 100 μg/ml) and then treated with or without LPS (1 μg/ml). Asterisks indicate the formation of hydroxyl radicals. Spectra are representative examples of three similar experiments.

Classical radical products such as nitric oxide and superoxide anions, which are generated upon macrophage activation, are associated with cell and tissue damage and redox signalling 24. To evaluate the efficacy of CME‐1 in reducing ROS formation in LPS‐activated macrophages, DCFDA, a cell‐permeative ROS‐sensitive dye, was used 16. Figure 7A illustrates that treatment with LPS (1 μg/ml) for 60 min. stimulated ROS generation significantly (2.3 ± 0.3‐fold, *P *<* *0.01, *n *=* *3) in macrophages compared with that of the control group. LPS‐stimulated ROS production in macrophages was reduced by the administration of CME‐1 (100 μg/ml) to 94.4% (Fig. 7B). Moreover, a typical electron spin resonance (ESR) spectrometry signal of hydroxyl radical formation was observed in LPS‐stimulated (1 μg/ml) cells compared with resting (phosphate‐buffered saline (PBS)‐treated) cells (Fig. 7C, traces a and b). CME‐1 (100 μg/ml) significantly attenuated hydroxyl radical formation in RAW 264.7 cells stimulated by LPS (1 μg/ml) (Fig. 7C, trace c).

### Ceramide signalling pathway and anti‐oxidative property expressed in the inhibition of CME‐1 on LPS‐activated primary peritoneal macrophages

**Figure 8:**
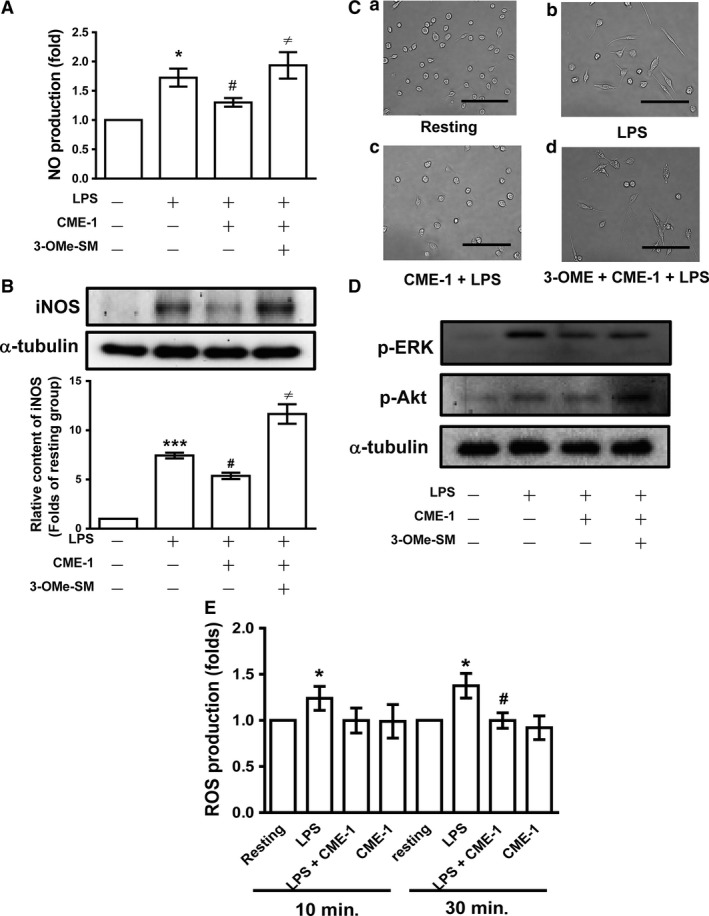
Expression of ceramide signalling pathway and anti‐oxidative property in the inhibitory mechanisms of CME‐1 in lipopolysaccharide (LPS)‐activated primary peritoneal macrophages. Primary peritoneal macrophages were pre‐treated with 30 μM 3‐OMe‐SM and then treated with CME‐1 (100 μg/ml), followed by LPS (1 μg/ml). (**A**) Nitric oxide production; (**B**) iNOS expression; (**C**) morphological change; (**D**) ERK and Akt phosphorylation; and (**E**) reactive oxygen species (ROS) production were determined as described in the Methods. Data are presented as the mean ± S.E.M. (*n *=* *3). *P <* *0.05 and ***P <* *0.001, compared with the resting group; ^#^ *P *<* *0.05, compared with the LPS group; ^≠^ *P *<* *0.05 compared with the LPS + CME‐1 group.

In addition, we further determined the inhibitory effects of CME‐1 in primary peritoneal macrophages stimulated by LPS. As shown in Figure 8A and B, CME‐1 (100 μg/ml) significantly reduced nitric oxide production as well as iNOS expression in LPS (1 μg/ml)‐activated peritoneal macrophages. LPS (1 μg/ml)‐stimulated irregular morphology with pseudopodia formation of peritoneal macrophages was abolished by the addition of CME‐1 (100 μg/ml) (Fig. 8C). Moreover, the pre‐treatment of 3‐OMe‐SM (30 μM) significantly reversed CME‐1 (100 μg/ml) suppressed nitric oxide production, iNOS expression, morphological change and the phosphorylation of ERK and Akt in peritoneal macrophages stimulated by LPS (1 μg/ml) (Fig. 8A–D). On the other hand, in primary peritoneal macrophages, CME‐1 (100 μg/ml) also revealed potent anti‐oxidative property against LPS‐induced ROS formation (Fig. 8E).

## Discussion

In Asia, *Ophiocordyceps* species are widely used in herbal medicines to treat respiratory, hepatic, kidney and inflammatory diseases. Polysaccharides extracted from *Ophiocordyceps* species possess immunoregulatory, antitumour and anti‐inflammatory properties 8, 9. Wang *et al*. 11 isolated CME‐1, a novel water‐soluble polysaccharide, from *Ophiocordyceps sinensis* mycelia. CME‐1 has been demonstrated to have anti‐ROS, antitumour and antithrombotic activities 11, 12, 13. Here, we demonstrated that CME‐1 has a potent inhibitory effect on nitrite formation and iNOS expression in LPS‐stimulated RAW 264.7 cells and primary peritoneal macrophages. As essential factors in inflammatory responses, iNOS and subsequent excess nitric oxide production are associated with the pathologies of numerous inflammatory diseases 25. The marked effect of CME‐1 on iNOS suppression indicates that CME‐1 is a potential therapeutic agent for inflammatory diseases. CME‐1 also did not exhibit cytotoxicity towards CME‐1‐treated macrophages. In addition, Wang *et al*. 11 indicated that CME‐1 has a cytoprotective effect against oxidative stress in hydrogen peroxide‐treated macrophages. Because macrophages are indispensable in immune responses, these results may also imply that CME‐1 exhibits immunomodulatory activity.

The acute inflammatory response of macrophages is an essential part of the host defence and innate immunity. Macrophage activation is stimulated by receptors that recognise microbial components, which subsequently activate inflammatory signalling cascades 3. Stimulating macrophages with LPS, the most potent immunostimulant of all bacterial cell wall components, elicits various signalling events through TLR4 activation for macrophage effector functions 4. Numerous studies have indicated that the activation of NF‐κB, MAPK and PI3K/Akt signalling pathways plays crucial roles in TLR4‐activated macrophages and gene expression 5, 6, 19. We determined the effects of CME‐1 on IκBα degradation and p65, Akt and MAPK phosphorylation in LPS‐stimulated RAW 264.7 cells. CME‐1 treatment generally suppressed IκBα degradation and protein kinase phosphorylation in LPS‐stimulated RAW 264.7 cells. Protein phosphorylation is a reversible process that is balanced by protein kinases and phosphatases 20. We thus determined whether protein phosphatase regulation causes the CME‐1‐induced comprehensive abolishment of protein kinase phosphorylation.

PP2A serves a leading proportion of mammalian phosphatases in cells, and it constitutes up to 80% of serine and threonine (Ser/Thr) protein phosphatases. This principal class of Ser/Thr protein phosphatases plays critical roles in modulating development, cell death and various signalling pathways 26. Additionally, PP2A modulates IκBα degradation and p65, Akt and MAPK phosphorylation 27, 28, 29. Therefore, we postulated that PP2A is involved in the inhibitory effects of CME‐1 on these pro‐inflammatory signalling pathways. OA is a representative diarrhoeic shellfish poisoning toxin and potent PP2A inhibitor 22. In the present study, OA pre‐treatment significantly reversed CME‐1‐suppressed iNOS expression; IκBα degradation; and p65, Akt and MAPK phosphorylation in LPS‐stimulated RAW 264.7 cells. These data imply that PP2A may be an essential part of the inhibitory mechanisms of CME‐1 in stimulated macrophages. To further determine the effect of CME‐1 on PP2A in macrophages, we examined PP2A activity using an immunoprecipitation phosphatase assay kit. In addition, the reversible methylation of Leu309 in a conserved TPDYFL motif of the carboxyl‐terminus of PP2Ac was known to regulate phosphatase activity and holoenzyme assembly 26. We thus evaluated dimethyl‐PP2A expression in CME‐1‐treated RAW 264.7 cells. CME‐1 treatment up‐regulated PP2A activity and dimethyl‐PP2A expression in RAW 264.7 cells, and OA preincubation significantly reversed CME‐1‐enhanced PP2A activation.

Three major families of endogenous molecules regulate PP2A activity, namely polyamines, metal cations and ceramides 30. Ceramides are a class of sphingolipid metabolites and have been reported to play a critical role in regulating immunity and inflammation 31. PP2A is a primary target for ceramide regulation. A recent study indicated that ceramide directly binds to SET, an inhibitory protein of PP2A, relieving its inhibitory action and thus increasing PP2A activity 32. According to knowledge gained in this emerging field, FTY720, a sphingosine analogue, was developed as an immunosuppressive agent and was also found to initiate PP2A activation 33. In the present study, pre‐treatment with 3‐OMe‐SM significantly abolished CME‐1‐induced PP2A activation and suppression of iNOS expression; IκBα degradation; and p65, Akt and MAPK phosphorylation in RAW 264.7 cells and primary peritoneal macrophages stimulated by LPS. Furthermore, CME‐1 significantly increased ceramide formation in LPS‐activated macrophages. These data elucidate a critical role of ceramide‐activated PP2A signalling in the inhibitory mechanisms of CME‐1 in acute inflammation. However, Wang *et al*. 11 indicated that the pre‐treatment of CME‐1 revealed cytoprotective effect on macrophages through the inhibition of H_2_O_2_‐induced tremendous ceramide formation. In the present study, ceramide increased within short‐term treatment of CME‐1 in LPS‐activated macrophages. On the other hand, in the study of Wang *et al*. 11, the significant inhibition of ceramide formation by CME‐1 occurred in macrophages treated with H_2_O_2_ for 6 hrs. These results may imply that CME‐1 has distinct activity on ceramide formation in different pathological conditions.

Oxidative stress is a harmful consequence of inflammation because irreversible oxidative cell or tissue damage may be associated with various diseases 24. The term ‘oxidative stress’ has been defined as an imbalance in the pro‐oxidant and anti‐oxidant systems. Pro‐oxidants, including superoxide anions; hydrogen peroxide; hydroxyl, alkoxy and peroxy radicals; and peroxynitrite, are generally considered ROS 34. Upon pathogen recognition, macrophages produce ROS through NADPH oxidase (NOX) activation. This oxidative burst may be involved in cell and tissue damage, pathogen killing and inflammatory signalling 35. Furthermore, up‐regulation of anti‐oxidant enzymes was found to suppress nitric oxide production or NF‐κB activation in macrophages 24. NOX‐dependent ROS production was associated with the stimulation of NF‐κB and p38 signalling, which is required for LPS‐induced activator protein 1‐driven transcription 36. In the present study, we determined the effects of CME‐1 on ROS formation in LPS‐stimulated RAW 264.7 cells and primary peritoneal macrophages using a DCFDA assay and ESR spectrometry. Treatment with CME‐1 significantly abolished LPS‐induced ROS production in macrophages. These results indicate that treatment with CME‐1 can relieve inflammatory diseases by reducing oxidative stress and its related inflammatory signalling.

Uncontrolled inflammation leads to various pathological conditions such as rheumatoid arthritis and sepsis 7. Developing new anti‐inflammatory treatments is therefore necessary for improving therapy for inflammatory diseases. Our findings demonstrate that CME‐1 significantly reduced iNOS expression in LPS‐activated macrophages by up‐regulating the ceramide‐PP2A signalling pathway and suppressing ROS formation (Fig. 7D). In conclusion, CME‐1 is a potential therapeutic agent for treating and preventing inflammatory diseases.

## Author contributions

Joen‐Rong Sheu, Ming‐Jen Hsu, Chih‐Hao Yang and Cheng‐ying Hsieh conceived and designed the experiments. Zhih‐Cherng Chen, Kuo‐Wei Jung, Wei‐Fan Wu, Ruei‐DunTeng and Szu‐Han Pan performed the experiments and analysed the data. Cheng‐Ying Hsieh wrote the manuscript. The authors all approved the final version.

## Conflict of interest

All authors declare that they have no conflict of interests.
